# Supplementary material for: Regenerative and Anti-Inflammatory Potential of Regularly Fed, Starved Cells and Extracellular Vesicles In Vivo
Source: Cells. 2022 Aug 30;11(17):2696. doi: 10.3390/cells11172696 (PMC9455002; doi:10.3390/cells11172696)
Supplement: Supplementary file 1 [file cells-11-02696-s001.zip › cells-1853936-supplementary.pdf]

Regenerative and Anti-Inflammatory Potential of Regularly Fed, Starved Cells and Extracellular Vesicles in Vivo

Federico Ferro [1,2,*,†], Renza Spelat [3,†], Georgina Shaw [2], Cynthia M. Coleman [2], Xi Zhe Chen [2], David Connolly [4], Elisabetta M. F. Palamá [5], Chiara Gentili [5], Paolo Contessotto [6,‡] and J. Mary Murphy [2,‡]

[1] Department of Medical, Surgery and Health Sciences, University of Trieste, 34125 Trieste, Italy
[2] College of Medicine, Nursing and Health Science, School of Medicine, Regenerative Medicine Institute (REMEDI), National University of Ireland Galway (NUI Galway), H91 W2T9 Galway, Ireland
[3] Neurobiology Sector, International School for Advanced Studies (SISSA), 34136 Trieste, Italy
[4] Discipline of Biomedical Engineering, School of Engineering, College of Science and Engineering,
National University of Ireland, H91 TK33 Galway, Ireland
[5] Department of Experimental Medicine (DIMES), University of Genoa, 16132 Genoa, Italy
[6] Department of Molecular Medicine, University of Padova, 35122 Padova, Italy
* Correspondence: ferro.federico@libero.it
† Authors contributed equally to the study.
‡ Authors contributed equally to the study.

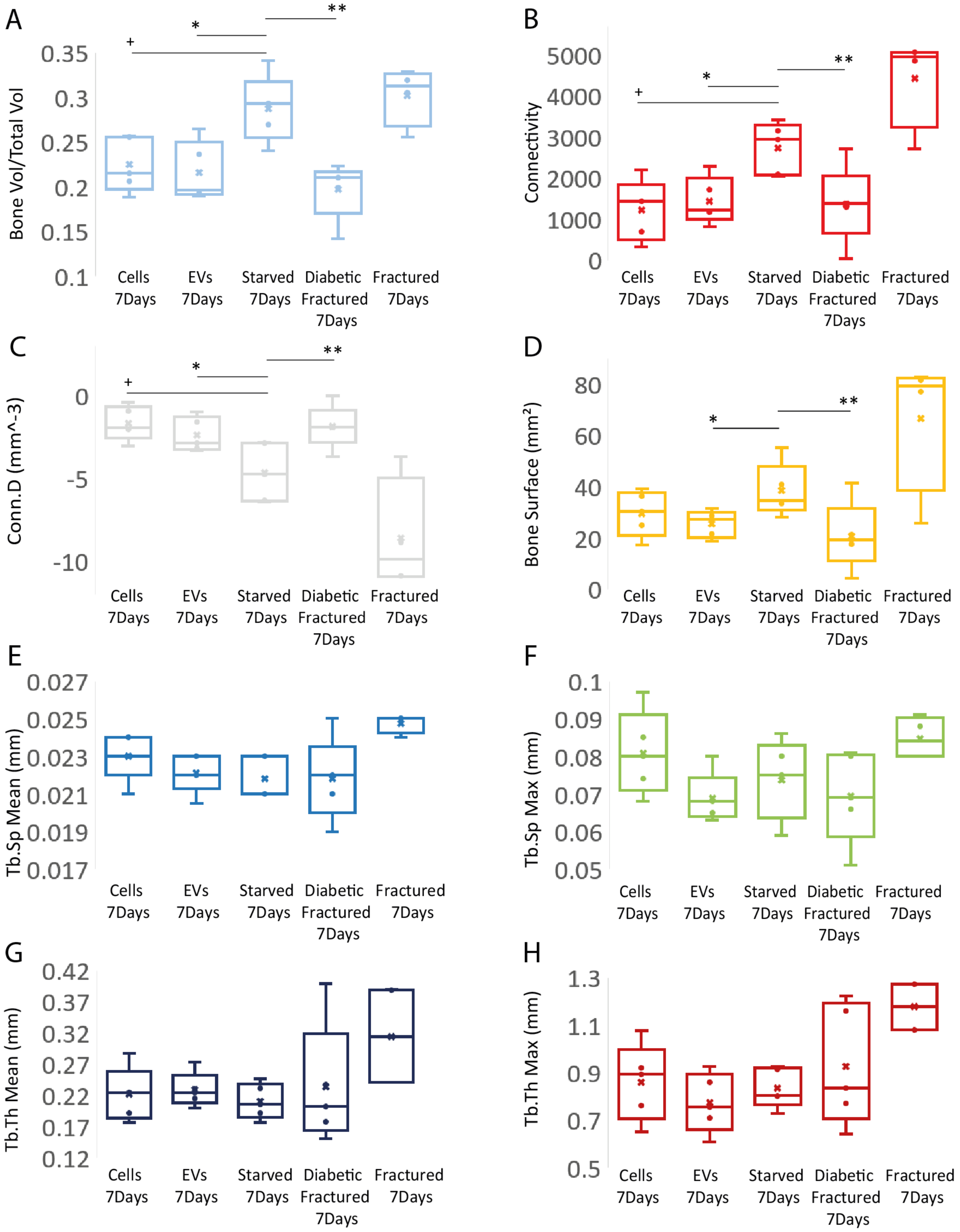

Figure S1. Analysis of the newly formed mineralized tissue five days after cells (starved and regularly fed) and EVs injections at the fracture site. (A-D) With respect to the diabetic fractured mice, preconditioned/starved cells induced a significant improvement in BV/TV, connectivity, Conn.D (mm^-3) and BS (mm²). (E-H) No significant difference was seen between the treatments for trabecular Tb. Th. Mean (mm), Tb. Th. Max (mm), Tb. Sp. Mean (mm) and Tb. Sp. Max. Results are presented as mean ± SD. p≤0.05 *respect EVs, +Cells, **Diabetic fractured (Sample size four to five animals/group, one-way ANOVA followed by Fisher's post hoc test). Abbreviations: regularly fed cells (Cells); starved cells (Starved); extracellular vesicles (EVs); bone volume/total volume (BV/TV); connectivity density (Conn.D (mm^-3)); bone surface (BS(mm²)); trabecular thickness mean (Tb. Th. Mean (mm)); trabecular thickness max (Tb. Th. Max (mm)); trabecular spacing mean (Tb. Sp. Mean (mm)); trabecular spacing max (Tb. Sp. Max (mm)).

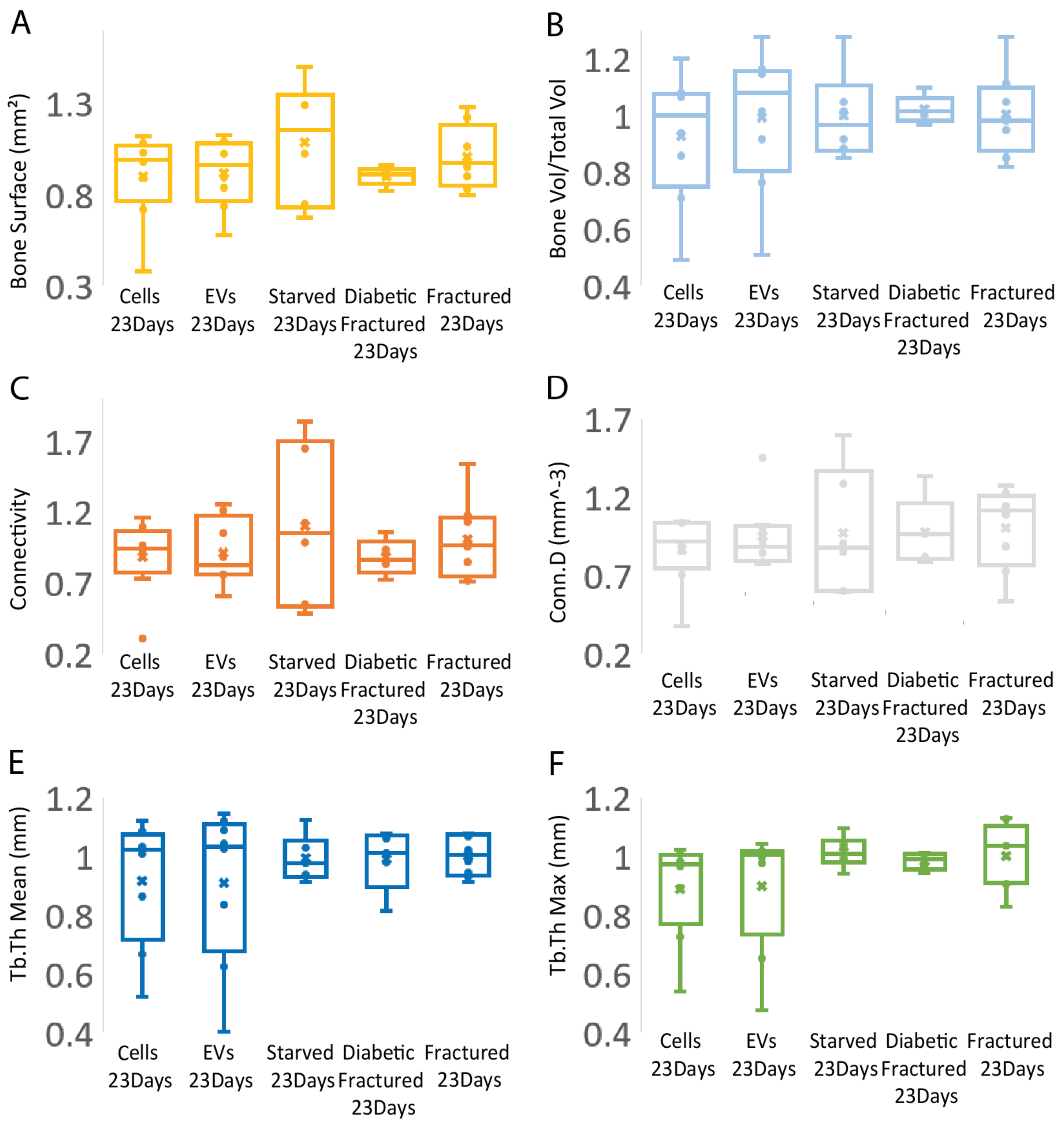

Figure S2. Analysis of the newly formed mineralized tissue at 21 days after cell and EVs injection at the fracture site. (A-F) With respect to the diabetic fractured mice, preconditioned/starved cells induced no significant improvement in BV/TV, connectivity, Conn.D (mm^-3) and BS (mm²), trabecular Tb. Th. Mean (mm), Tb. Th. Max (mm) at the later time-point (23 days) as evident by µCT parameters. The results are presented as mean ± SD. (Sample size five to eight animals/group, one-way ANOVA followed by Fisher's post hoc test). Abbreviations: regularly fed cells (Cells); starved cells (Starved); extracellular vesicles (EVs); bone volume/total volume (BV/TV); connectivity density (Conn.D (mm^-3)); bone surface (BS(mm²)); trabecular thickness mean (Tb. Th. Mean (mm)); ($p \leq 0.05$); trabecular thickness max (Tb. Th. Max (mm)); trabecular spacing mean (Tb. Sp. Mean (mm)); trabecular spacing max (Tb. Sp. Max (mm)).
